# Lifespan Development Seen through Niche Construction Theory

**DOI:** 10.1007/s12124-023-09806-8

**Published:** 2023-09-26

**Authors:** Bo Allesøe Christensen

**Affiliations:** Aalborg University, Aalborg, Denmark

**Keywords:** Nature, Normativity, Niche construction Theory, Odling-Smee, F. J.

## Abstract

I will here pick up on a suggestion made by Greve (2023) in this journal, namely that a proper understanding of lifespan development means defending a non-reductionist psychology taking biological processes seriously, but without reducing psychology to physiology. I will here suggest and argue for the use of niche construction theory as a way of providing a psychological theoretical perspective on lifespan development broad enough to contain both naturalistic and normative elements in a non-reductionist manner.

a man's reach should exceed his grasp – Robert Browning

Greve’s article (2023) opens up for interesting discussions in understanding some philosophy of science issues in psychology. I will pick out one here, which I believe is at the core of some of the worries and solutions that Greve presents in his discussion of the concept of lifespan. The issue turns on relating naturalism and normativity, which are two perspectives or approaches that seems, in one way of another, to lurk in the background when we speak of, for example, biological versus social psychology, or neurological versus cultural psychology. At times these perspectives seem antagonistic, especially in the fights for defining what the “right” psychology to pursue in university departments is. We might, of course, just bypass the issue, turning a blind eye to it, but then we misrecognize their interrelation, co-constitution or interdependence. Because the normative side is obviously dependent upon the natural side, without any life there will be no thought or understanding about it. And the other way around, without any concepts, language, models, theories etc., it would be difficult to understand how the piece of nature we call humans can come to an understanding of themselves and their surroundings as the sentient beings they are. As Harré has argued for a long time, and Greve emphasizes as well, the matter might just as well turn on whether these two “gestalts” of psychology can be reduced to one another, and if not – the most likely scenario – how, then, are we to understand their relation? As stated in Greve’s conclusion the challenge is combining “…the defense of a non-reductionist psychology that takes biological processes seriously without reducing psychology to physiology”, and in the same place he emphasizes that the potential solution lies in the development of abstract theory and not only through a physiological description of how human lives are realized. I will pick up on that here, by presenting some recent theoretical development addressing this thematic.

I will start by presenting the challenge of aligning the natural and normative by paraphrasing how the two American philosophers Wilfred Sellars and John McDowell conceive of it. I will hereafter relate this briefly to some of the research issues surrounding the concept of lifespan development according to Greve. Based on this I will suggest a way of combining the normative and natural, by using Niche-Construction Theory as a frame and illustrate this through some examples.

## Sellars and McDowell: the Challenge of Reconciling Two Pictures of the World

The American philosopher Wilfred Sellars made a distinction between two images of the world: a manifest image of human beings in the world and a scientific description of them. The manifest image is “the framework in terms of which man came to be aware of himself as man-in-the-world” (1991, 6), i.e. the framework in terms of which we ordinarily observe, understand and explain our world. This ordinary world consists fundamentally of persons, other living creatures and objects we engage with. Furthermore, emphasis is on *persons*, as language-using and reason-giving animals. Thus, within the manifest image, people are understood as *normative* sentient beings, i.e. they *think* and do things for reasons, i.e. “…only within a framework of conceptual thinking in terms of which [they] can be criticized, supported, refuted, in short, evaluated” (1991, 6).

In continuation of the manifest image, but also in opposition to it, is the scientific image. This image consists basically of the physical sciences’ description of the world, i.e. from the point of view of physics, chemistry and biology. For Sellars “…the scientific image presents itself as a *rival* image. From its point of view the manifest image on which it rests is an ‘inadequate’ but pragmatically useful likeness of a reality which first finds its adequate (in principle) likeness in the scientific image” (1991, 20) For the scientific image human experience, thought and action are explicable using theoretical terms taken from the relevant natural sciences. It thus reduces investigations into human beings to a matter of natural scientific explanations. From the manifest side, however, scientific understanding is rationally explicable as an achievement of human understanding. Thus, the manifest side is not seeking to reduce the scientific image but is emphasizing its meaningfulness to people through a shared commitment to think and act in empirically accountable terms.

Thus, both images claim an autonomy and a completeness of their respective understandings of the world, and the challenge, as Sellars saw it, and many people still see it, was reconciling both images in a synoptic vision. We can perhaps formulate the challenge using some concepts from John McDowell’s Sellars inspired book, *Mind and World,* of avoiding a baldly naturalism on the one hand, and a frictionless spinning in the void on the other hand (McDowell, 1994). If we reduce all human experience, thought and action to capabilities to be explained in natural scientific terms, then we fail to understand that human experience, thought and action is fraught with a normativity not explicable in selfsame natural scientific terms. All human sciences develop because of argument, of understanding what scientists say and do by placing these doings and sayings within a space of giving and asking for reasons. Reducing this space would be undermining the uniqueness, the normative authority, of the practices of sciences. It would make scientific descriptions and explanations into causal determined noise, rather than meaningful and justified claims. On the other hand, if we disregard a commitment to getting the world right, so to say, of accepting that there is a distinction internal to our practices between how the world is and how we understand it, then we fail to establish a sense of constraint on our experience, thought and actions. We end up in what McDowell terms a frictionless spinning in the void, where “nature” through basic empirical relations, does not play any role in constraining our normatively structured practices of giving and asking for reasons. So, in a somewhat trite phrase, nature without normativity is blind, but normativity without nature is empty. We need both to achieve a thorough understanding of human experience and psychological development.

I think we can see this tension of committing ourselves to letting constraints play a role in our scientific practices in Greve’s paper, especially in the development of a scientific perspective on developmental psychology, incorporating the whole of a life in a life span, and not just focusing on parts of the life span. Related to this is also the understanding of the relation between development and stability as a continuous relation through the lifespan and not seeing the lifespan as defined by increasing stability. Now, part of the crucial challenge trying to present a non-reductionst version of naturalism as a scientific perspective, is figuring out what kind of concept can encompass the bio-normativity of human beings. There has been a plethora of suggestions the last 20 years, the discussions of which will be left for another occasion. Instead, I will suggest that one way of addressing a non-reductionist conception of the life-span perspective, is by engaging with the notion of niche-construction. This is a complex issue, with many historical precursors^1^ and we can therefore only scratch the surface here by introducing, in the next section, two basic issues related to it. Niche-construction takes it departure in *life* as a dynamic biological and socio-cultural interaction between humans and their *umwelts*, and niche-construction theory understands this interaction as being adaptive as well as constructive. Hence, within this perspective life-span development could be understood as a continuous (re)construction of one or more niches, comprised of biological and socio-cultural elements, and as a response to changing biological and socio-cultural circumstances.

## Life-span Development as Continuous Niche-construction

The two basic issues alluded to above is, first, what the idea of niche construction is and implies, and two, the related notion of ecological inheritance, indicating how development is dependent upon both biological, environmental and socio-cultural heritage.

### Niche-construction Theory

While the notion of niche has a long history within ecology and ecological psychology, niche construction theory (NCT) was coined by Oxford biologist John Odling-Smee in the late 1980s as a way of understanding evolutionary processes differently than the conventional view on evolution (see Odling-Smee et al., 2013). The conventional view of evolution understands species as having the characteristics they have, enabling them to survive and reproduce in the best possible way, as an act of natural selection. NCT in contrast, emphasizes the capacity of organisms to modify environmental states, “Niche construction is the process whereby organisms, through their metabolism, activities, and choices, modify their own and/or each others’ niches” (Odling-Smee et al., 2013, 220) Hence, the focus is on the transformation or modification of organisms’ developmental and selective environment through their ongoing and cumulative interactions with that environment. In this sense “…animals manufacture nests, burrows, holes, webs, and pupal cases; algae and plants change levels of atmospheric redox states, and influence energy and matter flows by modifying nutrient cycles…” (Flynn et al., 2013, 296). And more specific examples pertaining to humans could be how “rice farming in the Neolithic may have contributed to selection of the copy number of gene AMY1, responsible for salivary amylase, which breaks down starch into simple sugars…In contrast, the contemporary Balinese cultural practice of irrigation systems used in rice farming provides a selective environment affecting cultural change in self-governing assemblies and religious beliefs” (Kendal, 2011, 242) Thus, there is a complicated reciprocal relationship between the genetic make-up of humans, the environment they live in and the socio-cultural practices they enact.

Unlike the traditional focus on natural selection, and because NCT recognizes that organisms can modify selection pressures in their own and other species’ environments, the organisms cannot be understood as passive victims of selection (Flynn et al., 2013, 298). In other words, organisms are to be understood as having a more constructive and active role in the shaping of their own and others evolution. This allows a dimension where changes occurring to the organism are not only external to the organism, such as environmental pressures, or internal to the organism but not under the organism’s control or awareness, such as genetic inheritance. Instead, changes are also seen as a result of human engagement, i.e. involving goal-directedness and active participation in the environments they partake but cannot fully control. As Gauvain claims this indicates the necessity of paying attention to and adopting a cultural-psychological notion of mind, i.e. “…mind as a symbol generating, meaning-making, artifact-devising, socially transmitting system that is simultaneously an individual, social, and historical (cross-generational) phenomenon.” (2000, 153) Thus the relations between human organisms and their environment, is not only co-causal but also normative. In Rouse’s terms, they enable a *non-dualistically naturecultural inferentialism*. Organisms are here understood more as goal-directed processes of environmental interaction than separate physical objects, and the biological environment “is not the entirety of their physical surroundings, but a pattern of affordances for and obstacles to that life-process.” (2018, 243) Developing and transmitting knowledge and meaning is therefore a crucial feature in constructing, selecting, and even destroying niches. Which brings us to the second issue.

### Ecological Heritage

The shaping of niches over time, and how the resources and conditions associated with this shaping are passed on to descendants, is called ecological inheritance (Odling-Smee & Laland, 2011). Examples of this can be schools designed for children to learn, or protective environments like nests or perambulators, for infants. Ecological inheritance is different than genetic inheritance, since It is transmitted through the modification of an external environmental and social transmitting system, and not by genetic reproduction. (ibid. 223).

What separates human niche constructions from animal niche constructions is, of course, the complexity of the human constructions, with languages and conceptual practices as preeminent to humans, but the creation of niches in outer space is ‘up there’ as well. And it is this active part, several authors – including Gauvain above – have emphasized as carrying a similarity to notions of human agency presented in the human sciences (Flynn et al., 2013, 298). Or in other words, human niche construction is socio-cultural, and human ecological inheritance consists therefore of a subset of socio-cultural inheritance besides the biological inheritance:

“…unlike genetic inheritance but like ecological inheritance, cultural inheritance is continuously transmitted by multiple human beings, to multiple other human beings, within and between generations, through an external environment, by a number of different routes, such as learning obliquely from the previous generation, learning horizontally from siblings, friends, or peers, copying the behavior with the highest payoff, or conforming to the majority behavior” (Odling-Smee & Laland, 2011, 227).

Thus, ecological inheritance can help account for Greve’s claim, that “…developmental dynamics implies a comparably functional realization of horizontal (learning) and vertical (inheritance) information transfer.” (p. 18). Furthermore, without reducing psychology to biology it allows for a non-reducible and more dialectic, i.e. capable of being in opposition as well as aligned, relationship between the biological and socio-cultural parts of ecological inheritance within NCT. But how can we then relate this to life-span development?

## Examples Indicating Lifespan Development within a NCT Perspective

In general, if we follow the approach to Niche-Construction and the idea of ecological inheritance presented above, then we need to see the three aspects inherited in conjunction: humans inherit and transform not only genetic information and cultural knowledge from the ancestors but also modified environments. Let us present some examples indicating how the concept of niche-construction can contribute to the understanding of life-span development.^2^ The first example, taking from Odling-Smee and collaborators, will focus on how the human modification of the external environment through the production of yams affects the genetic inheritance, while the second will trace the development of niches as part of children’s cognitive development. The second example will take its departure in Tomasello’s descriptions of what he and collaborators claim are a cognitive revolution occurring when infants are roughly around 9 months old. The point here will be understanding how this developmental revolution is connected to modifications in the physical environment made by the parents. These modifications create a scaffolding for the development of basic cognitive capabilities and the cultural transmission of knowledge as part of an ecological inheritance. In both cases a lot more could be elaborated on than space allows here.

### Production of Yams as Niche Construction

Odling-Smee et al (2013) present an example of yam cultivators in West Africa where an increase in the frequency of a hemoglobin allele causing sickle-sell anemia as an indirect effect of the yam cultivation was detected. "These people traditionally cut clearings in the rain forest to grow their crops, creating more standing water and increasing the breeding grounds for malaria-carrying mosquitoes. This, in turn, intensifies selection for the sickle-cell allele, because of the protection offered by this allele against malaria in the heterozygous condition “ (p. 252) Two points are important noticing as a result of this.

First, this could look like a clear relation between the development of new cultural practices which then affects the allele frequencies, hence what could be termed as a sort of gene–culture co-evolution. But for Odling-Smee et al. this would be missing the central point that the crucial factor is the amount of standing water occurring in the environment *as a result* of the cultivation. This is an ecological and not just cultural variable depending also on factors, like rainfall or flooding, not controllable by the yam cultivators. Hence, a suggested co-evolution of the genetic and cultural inheritance systems needs to be supplemented by this third ecological variable, the broader changes in the material surroundings, to fully understand what occurs within this yam production as a *niche*-construction.

Second, Odling-Smee et al., (2013, 262) describes the yam production as the creation of a cultural niche creating a modified environment which, under the right natural conditions, leads to new *natural* selection pressures, i.e. dealing with the mosquitos. Hence, what happened in the above example, was a change, a natural response or selection, in the form of an adaption in the gene pool of the people involved. But, and this is their point, if the yam cultivators developed a medicine for dealing with the malaria, a different technology for draining the standing water, or new ways of cultivating the yam not diminishing the area of the forest, then this would rather count as *cultural* response or selection with the natural selection probably being unnecessary.

To reiterate, besides the genetic and socio-cultural inheritance we need to focus on the inheritance of a modified environment as well. I have already referred above to Greve’s point that developmental dynamics implies a realization of horizontal and vertical information transfer. We can perhaps express the contribution of understanding niches as developing a contextual sensibility towards the material conditions, i.e. genetics, environmental, artefactual and embodiment, tied to this information transfer and in a non-reductive sense. Niche-Construction as lifespan development would then focus on ecological inheritance comprising the horizontal and vertical axes but adding a focus on the modified environments wherein these axes are related.

### Niche Construction in the Development of Children’s Cognitive Capabilities

Another example where niche construction theory could contribute to our understanding of lifespan development is the revolution children undergo around 1 years of age. This revolution facilitates the development of their cognitive capabilities of understanding an independent reality and themselves and others as intentional agents, with both important factors for understanding the roots of human societal life. This example will not focus on the genetic inheritance like the example above. Instead, the focus will be on the cultural selections to modifications in the environment, what Gauvain above termed as the individual, social, and historical (cross-generational) part of ecological inheritance. So, here it will just be presumed that there is a genetic inheritance from parents to children, and also that different biological environments will have different effects on children possibly impairing or advancing their cognitive development.

Building upon and developing Piaget’s work (Piaget, 1952) on the development of children, and especially on the ideas of object permanence and intentional and planned behaviour, several researchers – including Tomasello – have suggested that our common sense metaphysical worldview and social psychological framework is established already in early age. Based on several investigations (eg. Carpenter et al., 1998; Tomasello, 1999), Tomasello has indicated that a revolution occurs around 9 months with infants moving from a dyadic to a triadic relationship with the world. The newly born babies already show signs of being very social creatures capable of recognizing other persons as animate beings different from physical objects. They engage, for example, in proto-conversations with their caregivers, i.e. social interactions between parent and infant with acts of reciprocal attention established through looking, touching or vocalizing thereby expressing and sharing emotions. Furthermore, they mimic movements of the adults, and especially movements of the mouth and head, with some researchers claiming that this indicates the beginnings of a process of identification with conspecifics (Tomasello, 1999, 59–60). This process of social attunement, with an incipient understanding of turn-taking and the establishing of processes of identification, is, until the age around 9 months, primarily dyadic, “If people are around when they are manipulating objects, they mostly ignore them. If objects are around when they are interacting with people, they mostly ignore them.” (1999, 62) Thus, in this first dyadic relationship between infant and surroundings, a rudimentary sense of sociality, distinguishing between objects and persons and relating to the latter in specific ways, seems to exist. Also, a basic understanding of the physical environment – established through basic movements and manipulation of objects – seems to be in place. This changes, according to Tomasello, to a more triadic relationship based on the infants understanding their worlds in new ways.

At about nine months infants begin to engage in joint attentional activities indicating the beginning of an understanding of other persons as intentional agents whose relations to objects or other people may be followed, directed, or shared. This is triadic since it involves a coordination of the infants’ interactions with objects and people, creating a referential triangle of child, adult and object or event towards which attention is shared. According to Tomasello, at this age,

“…infants for the first time begin to flexibly and reliably look where adults are looking (gaze following), to engage with them in relatively extended bouts of social interaction mediated by an object (joint engagement), to use adults as social reference points (social referencing), and to act on objects in the way adults are acting on them (imitative learning).” (1999, 62).

Furthermore, related to this and occurring around the same age, children begin to direct the attention of adults using deictic gestures such as pointing towards objects as independent entities. In addition to indicating a triadic relation, these deictic gestures have both imperative and declarative functions, attempting to get the adult either to do, or attend to something. Thus, along with the understanding of others as intentional agents, the infants begin to understand themselves as intentional agents as well. This sets in motion the creation of a personalized subjectivity through the socio-cultural relation to others, similar to the processes described by Vygotsky or G. H. Mead. According to Tomasello this triadic relation, then, serves as the ontogenetic background for the transmission of culture, because this basic collective intentionality opens the infants up to an intersubjectively shared meaningful reality filled with material and symbolic artifacts and social practices created by the members of their culture (1999, 91). While Tomasello claims the development from the dyadic to a triangular relationship is general for all human cultures, the way this is realized depends on the specific cultures. Furthermore, he follows Gauvain (1995) in calling this an ontogenetic niche for human development (Tomasello, 1999, 79) with infants and young children inheriting a whole way of living, a life-form, as well as receiving instructions from adults in acquiring skills and knowledge.

The movement from dyadic to a triadic relationship can therefore be described through the characteristics of transitions described by Zittoun et al. (2013, 263) as involving learning processes, identity changes and sense making. First, it involves the learning processes initiating the infants into different cultural practices depending upon the instructions from conspecifics (the space of reasons alluded to in the beginning). This also includes the social learning of handling of tools and artifacts through a) stimulus enhancement, where an adult picks up an object making the infant interested in touching and handling the object; b) emulation learning, where an infant learn new uses of an object watching an adult manipulating this object and c) imitative learning, where the child learns something about intentional action (Tomasello, 1999, 81) Second, it establish rudimentary forms of understanding identity changes by beginning to build up membership categorizations by distinguishing between objects and persons, as well as developing personal (I, me and you) and collective identities (we, us and them). Third, it involves sense making processes connecting previous with new experiences, and the move to a triadic relationship paves the way to understanding and using narratives as part of this sensemaking (Bruner, 1990).

Based on this very brief and overall description of the developmental change from a dyadic to a triadic relationship, let us return now to the niche construction theory described above. Tomasello’s description – as well as the development process described by Zittoun et al. – can be complemented by a focus on how the concrete environment is both inherited and modified, thereby influencing these developmental and transitional processes. I will give one example here – the transportation of infants – trying to indicate how this could be understood moving from the dyadic to a triadic relationship using niche construction theory. To reiterate I will not focus on the physical development of a child being a result of the interplay between genetic inheritance and material circumstances in which a child is brought up.

First, let us notice that, as Wall-Scheffler et al (2007) claim, the emergence of means for the carrying of infants probably is related to the emergence of bipedalism in human evolution, with carriers like the baby sling facilitating the possibility of using the hands, moving over a longer distance, and escaping predators instead of the exhausting effort of carrying the infant in the arms. Already Sewell (1923) drew attention to the many different cultures using different kinds of paraphernalia for carrying infants. Thus, ancient artwork depicting the use of slings exist in many cultures, and other means such as the cradleboard has been widely used as well. The use of slings or cradleboards usually stops – or at least slows down – when the infant has developed the necessary sensory-motor skills to walk. However, due to different natural selection pressures some cultures develop niches where the use of a carrier persists for much longer periods than other cultures.

One example of this is the Ache people in Eastern Paraguay. As described by Kaplan and Dove (1987) this is a highly nomadic people spending significant amounts of time making trips into the subtropical broadleaf evergreen forest foraging. During these trips the Ache move camp daily, and because of this, mothers and their children spend basically all their time in uncleared or partially cleared spaces. Even when sleeping they clear only an area big enough for sleeping sites. The women spend all their time caring for the children, and due to the natural hazards in the forest, “Children younger than three years of age rarely venture more than a meter from their mothers and spend some 80–100% of the time in tactile contact with them.” (p. 191). During their investigations Kaplan and Dove discovered – what we can call a prolongation of the dyadic period – that Ache parents were very reluctant to allow their children, less than two years, to explore the environment. While this influenced the development of the sensory-motor skills, the children did not begin walking more than a few steps without falling until they were 2–3 years, Kaplan and Dove also suggests that this ‘prolongation of the dyadic period’ seems to delay their language and productive skills in comparison to other children. In other words, these children moved from the dyadic to the triadic relationship later in their development, due to their mothers constructing a safe niche, carrying and keeping the children close while moving from place to place, as a response to the natural but hazardous selection pressures^3^.

Second, let us take a (theoretical and based on personal experience as well) look at a modern and western phenomenon of child transportation, namely the perambulator or pram. Though also appearing in other cultures, again see Sewell (1923), the specific western one was invented in the middle of the 18 century (Morgan, 2022). It was invented by William Kent around 1733 for the Duke of Devonshire and was designed to be pulled by a goat or small pony. The first patent for a pram was received by Charles Burton in 1853, and by that time the pram had already evolved from a 4-wheeler to a model resembling contemporary strollers with only three wheels. For safety and comfort reasons springs and upholstery as well as breaking mechanisms were also introduced. During WW2 gas-safe prams were even introduced with the carriage having a metal lid containing a ventilation devise and a glass window so the baby could see the parent and vice versa (Morgan, 2022, 14–17). The development of the pram coincided with the modern development of traffic systems. To be able to use a pram or a stroller as a safe means of transportation, sidewalks needed to be invented. Sewell suggest that at the time he publishes his history of the pram, around 3 million prams were in use in Britain (Sewell, 1923, 716). Combined this indicates that on a macro-level the part of human cultural niche construction we connect with concrete production and transportation systems was already developed. As Amato puts it “Both stroller and wheelchair testified not only the desire to move people about but also to the existence of relatively level surfaces on which to do it.” (2004, 237) The non-ambulatory purpose of mothers promenading their babies on streets, of course also reflected a culture were leisure and economic abundance were the (nichean) privileges of the few. And apparently these prams were not only a joyous means of transportation or ‘show-off’; the manufacturer Charles Burton moved his production to England from New York because of reports of how prams collided with pedestrians (2004, 311).

Now, I am going to suggest (again theoretically and based on experience) that the pram and stroller can be understood as having some sort of scaffolding function in the sense of the classic article by Wood et al. (1976). The difference being that the scaffolding function is here tied more specifically to the material conditions (the design of the carrier) and not only the function of the adult (the tutor for Wood, Bruner and Ross). Recall the difference between dyadic and triadic relationships for the infant. In the dyadic relationships the infant focuses the attention on an object or person and leaves the rest out. It is a focus aiming at an awareness and understanding of the features of the object in front of it, or for engaging regularly in the proto-conversations with adults. Now imagine a pram. It is designed in such way, that the child lies in the bassinet facing the adult pushing it. The upper section of the pram capable of being put up protecting the infant from the weather, is often decorated with toys and figures the infant can look at and touch. When walking with the pram the adult continuously shifts the attention between the road and talking and making faces to the child. Any stop to rearrange the interior of the pram, is often followed by a smile towards the infant and some words, with the child responding. In this way, even while walking, there is still a basic turn-taking between the adult and the child and if the upper section is up, it might help focusing, ‘tunneling’, attention towards the adult or the toys hanging down. When the child reaches an age where the sensory-motor skills are developed to such a degree that it can sit up in the pram (with support from the pram), then the most ritual dyadic movement – as Tomasello (1999, 62) terms it – of raising one’s arms above the head can be carried out. This request of wanting to be picked up is dyadic because no outside object is involved; it is imperative because it is about what the child express it wants, and furthermore it is ritualized because it is not an act of imitation. The pram therefore offers an additional scaffolding besides directing the attention, it helps the child signaling that it wants something to be done, establishing also a first sense of the child as an intentional agent.

Moving on to the stroller – of the umbrella, running or pushing kind – this seems to present a scaffolding supporting a more triangular relationship. Of course, there will be more intermediate cases where the infant can sit in the pram without support, or even a carrying sling with the infant facing the adult carrying it. Here it will be possible to direct the infant’s attention to outside objects, through gestures like pointing accompanied and supported by exclamations like “Look, it’s a dog”. However, with the stroller the design is different. Here both the child and the adult are facing forward when walking. This indicates a scaffolding where the attention directed to the surroundings becomes more important, and thereby supporting the possibilities of learning (stimulus, emulation, imitation) connected with the triadic relationship. For example, while walking the child gestures towards what it sees, for example by pointing or imitating what it sees and hears, with the parent reacting to the child by recognizing the gestures “Yes, this is a nice dog”, or correcting the child if it like reaches for the dog. Thus, using the stroller as a means of transportation can be understood as scaffolding the creation of shared social experiences, “we said hello to a dog today”, with possible cognitive and emotional features, “It was a bulldog, and she was a little afraid of it”. Furthermore, an additional conjecture will be that it also supports the child’s initiation into aspects of the normativity of culture, learning how to behave and not behave around dogs (the space of reasons). The stroller therefore unlike the pram, seems to provide a material support of a more triadic relationship between the infant and the world. Both by facing forward and thereby being directed to objects and persons in the surroundings, but also – by not directly seeing and interacting with the parent – triadic as sharing an experience *in* the world and *of* the world with the parent. In the world through the action of being strolled encountering new objects, persons and events, and of the world by gradually learning to articulate – by gestures or vocalized language – this experience being in the world, by connecting previous experiences to new ones.

The examples presented here indicate, as a start, how the niche construction theory can be connected to life span development, and especially the dynamics between rupture and continuation as well as cultural variation. My examples have focused primarily on the development of children, as an example of variation and continuity. In the opposite end of life, a study by Garvey and Miller (2021) indicates, among other things, the role that technology–in this case the smart phone–plays in a community in Ireland as a scaffolding device for the production and reproduction of triadic niches, between subjectivities, intersubjectivities and the objective environmental surroundings, when physical and mental health is deteriorating.

## Conclusion

In Greve’s conclusion he claims that the special potential of the theory of evolution lies in the abstract theory. The perspective of NCT as argued for here has such theoretical potential, and has been presented without touching upon the details of combining it with life-span development generally beyond the examples presented. The point has rather been to present a possible perspective on combining life span development with a non-reductive interpretation of biology. The argument has therefore centered more on how the dialectics of nature and socio-cultural normativity assumed in NCT could be one way forward allowing for an evolutionary approach to human development without succumbing to a version of natural reductionism.

Furthermore, and as indicated, NCT is broad enough to allow for variance and diversity in the construction of niches to reflect diversity across the life-span. But through the notion of ecological inheritance, it is also broad enough to allow for a sense of continuity. Thus, NCT holds a promise of understanding the relation between stability and change Greve claims is central to understanding life-span development and as involving a dynamic relation between natural and socio-cultural conditions. The relation here can hopefully reflect the intention of the Browning quote in the beginning, that our relationship with the surrounding world surpasses our grasp of it. That though our lives cannot be reduced to nature, our grasp of these lives will never be self-sufficient.

## Footnotes

1 Precursors could be the ecological based understanding of biology connected with for example Uexküll’s notion of *Umwelt* (Uexküll, 1909), or Gibson’s ecological psychology (Gibson, 1966) as opposed to scientific understandings reducing biology to laboratory investigations of brains and genes. See also Brinkmann et al. (2023).

2 Theories of lifespan development can, of course, also contribute to our understanding of niche constructions, a subject which will be left for another occasion.

3 As Kaplan and Dove also notes, at the age of 8 – 10 Ache children are no different from other children in terms of their development.
